# Engineering an indoleamine 2,3-dioxygenase immunotherapy *via* selective cysteine-to-serine mutations

**DOI:** 10.1039/d5me00106d

**Published:** 2025-09-19

**Authors:** Jennifer A. Simonovich, Arun Wanchoo, Ryan A. Clark, Junha Park, Ayumi Shigemoto, Benjamin G. Keselowsky, Gregory A. Hudalla

**Affiliations:** a J. Crayton Pruitt Family Department of Biomedical Engineering, Herbert Wertheim College of Engineering, University of Florida PO Box 116131 Gainesville FL 32610 USA bgk@ufl.edu ghudalla@bme.ufl.edu +1 (352) 392 9791 +1 (352) 273 9221 +1 (352) 273 5878 +1 (352) 273 9326

## Abstract

Indoleamine 2,3-dioxygenase is an immunomodulatory enzyme that shows great promise when delivered exogenously as a protein therapeutic. However, IDO activity is under complex redox control, mediated in part by multiple cysteine residues within its primary sequence. We have characterized three IDO mutants in which solvent-accessible cysteine residues were mutated to chemically-similar serine residues, “IDO_C4S4_” with C112S, C159S, C206S, and C308S mutations and “IDO_C5S3_” with C112S, C159S, and C308S mutations based on prior reports that C206 is necessary for catalytic function, and IDO_C0S8_, in which all cysteine residues were mutated to serines. IDO_C0S8_ was expressed in poor yield and demonstrated less than 1% activity when compared to wild-type IDO. In contrast, IDO_C4S4_ and IDO_C5S3_ demonstrated robust enzymatic activity, though IDO_C5S3_ had a slower *V*_max_ than wild-type and IDO_C4S4_. Computational predictions and experimental measurements suggested a high degree of structural similarity between the wild-type IDO and IDO_C4S4_, with subtle perturbation of α-helical content for IDO_C5S3_. The structure of IDO_C0S8_ was predicted to be significantly different than that of wild-type IDO. IDO_C4S4_ and IDO_C5S3_ were more stable than wild-type IDO over time at physiological, ambient, and reduced temperatures, likely due to diminished oxidation of the mutant IDO forms. Based on the increased *V*_max_ and robust thermal stability of IDO_C4S4_, we fused it to the anchoring moiety galectin 3, to evaluate its effectiveness in a mouse model of psoriasis. The IDO_C4S4_-galectin-3 fusion blunted the rate and severity of disease as compared to wild-type IDO-galectin-3 fusion. When compared to historical data with Cys-Ala IDO mutants, this study highlights the importance of employing amino acid substitution according to similarity in isosteric and isostructural shape to advance IDO as an immunomodulatory therapeutic.

## Abstract 2

Selective cysteine-to-serine mutations improves enzymatic stability and therapeutic efficacy of the immunomodulatory enzyme indoleamine 2,3-dioxygenase.

## Graphical abstract

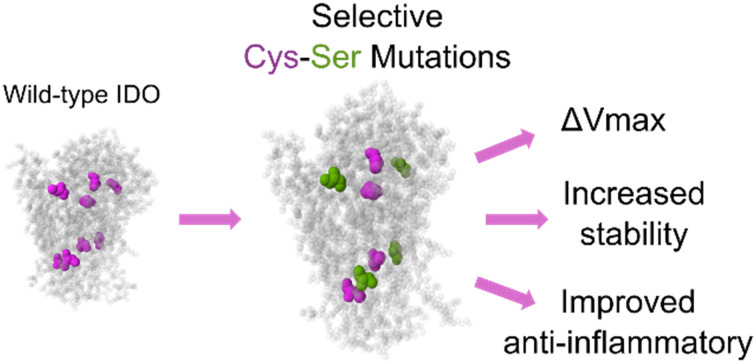

**Design, System, Application**

Enzymatic stability poses a considerable challenge to translation of biotherapeutics, as changes in conformational state can disrupt catalytic activity and functionality. If protein misfolding is driven by key amino acids, mutation into a more stable residue can reduce protein instability. We present a selective cysteine to serine mutation strategy for the immunomodulatory enzyme indoleamine 2,3-dixoygenase (IDO). By targeting only the most solvent accessible cysteines of IDO, the IDO mutants shared a high degree of secondary structure similarity, which in turn preserved enzymatic activity. These mutants also improved thermal stability during warm and cold storage, while also improving its therapeutic efficacy in a mouse model of psoriasis as compared to wild-type IDO. Computational predictions allowed for pre-screening of candidate mutants, where several key metrics predicted low enzymatic function in non-selective Cys-Ser mutations, which was seen after microbial expression. Not only does our work demonstrate improvements to a promising immunotherapeutic, but it also highlights the importance of employing amino acid substitution judiciously, and choosing replacement amino acids according to similarity in structural shape.

## Introduction

Indoleamine 2,3-dioxygenase (IDO), an enzyme catabolizing tryptophan,^1^ is an attractive immunoregulatory therapy. For example, IDO provided *via* overexpression in transferred cells can ameliorate type 1 diabetes^2,3^ and psoriasis.^4^ mRNA delivery of IDO has shown efficacy in the treatment of T cell mediated diseases.^5^ Exogenously supplied IDO protein confers dendritic cell resistance to LPS stimulation^6^ and is a robust anti-inflammatory.^7^ Yet, whether delivered as a gene or as protein, IDO enzymatic activity is under complex redox control.^1,8–13^ Immune cell activation in diseased tissue results in increased redox factor production, which can cause oxidative and nitrosative stress, redox signaling disruption, and molecular damage.^14,15^ IDO cysteine residues, of which there are eight, have been reported to contribute in part to enzymatic inactivation in high oxidant conditions.^8,16,17^

Cysteine residues often pose problems in protein therapeutic development. Cysteines can undergo oxidation into disulfides,^18^ which can lead to protein misfolding and aggregation.^19^ The sulfhydryls in cysteines can also oxidize into sulfinic, sulfenic, and sulfonic acids,^20^ which can disrupt co-factor binding (*e.g.*, metal ion coordination)^21^ and hinder substrate binding.^22^ Cysteine oxidation can furthermore change protein conformation, resulting in altered if not inactivated function.^23,24^ Given the complex character of cysteines, it can be advantageous to remove such residues, if possible to do so without significantly perturbing protein form and function.

In the case of IDO, the role of each of its eight cysteine residues is poorly defined. IDO does not contain any disulfide bridges, and cysteines in proximity to the heme active site are not thought to be directly necessary for enzymatic activity.^17,25^ Chemically modifying the cysteines of IDO can lead to inhibition. Selenyl–sulfide bonds formed between cysteine and the organoselenium drug ebselen inhibit IDO by changing the conformational state and disrupting enzymatic activity.^26^ Later work also showed multimerization and inactivation of IDO after oxidation through the formation of protein-centered radicals, though the specific amino acid residues involved have not yet been identified.^8^ Mutating cysteines to alanines can also reduce enzymatic activity. In particular, IDO activity decreased by more than 50% when C85, C206, C272, and C335 were individually mutated into alanines (Table S1).^27^

Mutating cysteines to alanines can result in unexpected changes to protein form and function due to the differences in their side-chain chemistry.^28,29^ In contrast, cysteine and serine are similar in isosteric and isostructural shape, differing only by atomic substitution of the sulfur in cysteine with oxygen in serine.^30,31^ Cys-Ser mutations have shown successes in engineering more stable protein therapeutics. In one instance, surface Cys-Ser mutants of galectin-1 resisted oxidative inactivation by eliminating susceptibility to covalent cross-linking, thus retaining activity in oxidative environments.^32^ Furthermore, Cys-Ser substitutions on the surface of IL-18 improved solubility and reduced aggregation.^33^ In contrast, in the copper binding proteins human ATP7B and *E. coli* CueR metalloregulator, Cys-Ser mutations were not tolerated at sites where cysteine coordinated copper ions and diminished enzymatic activity.^34^ Thus, judicious investigation of candidate mutation sites is necessary to balance protein biophysical properties for improved stability and function.

We investigated Cys-Ser IDO mutants to determine the impact of those mutations on enzymatic function, secondary structure, stability under different storage conditions, and therapeutic effectiveness. We limited mutations to C112, C159, C206, and C308, which are the cysteine residues predicted to be most solvent accessible (*i.e.*, on the “surface” of the protein) based on crystal structure and *in silico* predictions. We generated two mutants – IDO_C4S4_, where all surface cysteines were mutated into serines, and IDO_C5S3_, where C112, C159, and C308 were mutated into serines but C206 was maintained, based on a prior report suggesting its role in enzymatic function.^27^ We found that both Cys-Ser mutants generally retained IDO's enzymatic function and structure. IDO_C4S4_ was observed to be the most stable variant at reduced (4 °C) and body (37 °C) temperatures and outperformed wild-type IDO domain in the fusion protein IDO-galectin3 (IDO-Gal3) *in vivo* in an imiquimod (IMQ)-induced psoriasis mouse model.

## Experimental

### IDO cysteine mutants design and production

Selective mutations of cysteines into serines were formed at sites 112, 159, 206, and 308 for IDO_C4S4_ and at 112, 159, and 308 for IDO_C5S3_. Total mutation of cysteine to serine was formed at all sites for IDO_C0S8_. The genes encoding these mutant IDOs were inserted into pET-28a (+) vectors between NdeI and Xhol restriction sites, and were produced by and purchased from Genscript. The plasmids were then transformed into Origami B(DE3) *E. coli* competent cells (Sigma-Aldrich) for protein expression, with successful transformation confirmed by Sanger sequencing (Genewiz). Protein expression was performed using the autoinduction method.^35^ Bacteria were pelleted and lysed, and IDO was purified from the lysis supernatant through a HiTrap TALON cobalt column (Cytiva) and size-exclusion chromatography (GE Life Sciences). Purity was ensured by sodium dodecyl sulfate polyacrylamide gel electrophoresis (SDS-PAGE) and Coomassie staining. Yield for IDO_C5S3_ and IDO_C4S4_ mutants was ∼2.8 mg and ∼2.5 mg of protein per liter of growth volume, respectively. Concentration was determined *via* the Pierce Bradford Protein Assay Kit (ThermoFisher), prior to use. Absorbance spectra were captured by NanoDrop ND-1000 Spectrophotometer. Wild-type IDO production was performed following the same method as above. IDO-galectin3 (Gal3) fusion mutant were engineered through a combination of the IDO_C4S4_ mutant and our previously established production of IDO-Gal3.^7^ Yield for IDO_C4S4_-Gal3 mutant was ∼3.5 mg of protein per liter of growth volume.

### Enzymatic activity

Wild-type IDO_C8S0_, IDO_C5S3_, and IDO_C4S4_ were stored at 4 °C, room temperature, and 37 °C, and were sampled periodically to test for changes in enzymatic activity, with no additional steps taken to reduce oxidizing conditions. 17.88 pmoles of IDO was combined with tryptophan, catalase, and the electron donors methylene blue and ascorbic acid. The absorbance of *N*-formyl-kynurenine (NFK), a precursor to kynurenine, was read at 321 nm for ten minutes, and specific activity (unit of pmol NFK per min per pmol IDO) was determined from the slope of absorbance at 321 nm and the extinction coefficient of NFK, with a pathlength of 0.32 cm. Enzymatic activity for all timepoints were normalized against *t* = 0 IDO activity, and activity half-life was calculated *via* best fit. The kinetic parameters of wild-type IDO_C8S0_, IDO_C5S3_, and IDO_C4S4_ were determined by the Michaelis–Menten equation after varying tryptophan concentrations. Activity half-life and IDO kinetic parameters were compared using a one-way ANOVA and Tukey's multiple comparison test (Graphpad Prism).

### In silico analysis

Predicted secondary structures of wild-type IDO_C8S0_, IDO_C5S3_, and IDO_C4S4_ were generated using AlphaFold v3.^36^ Structural similarity was assessed by rigid FATCAT pairwise structural alignment^37,38^ (PDB) comparison of root mean square deviation of atomic positions (RMSD) and template modeling (TM) score. Predicted secondary structure composition for each protein was calculated using STRIDE,^39^ and then percentage of each structural component was compared between wild-type IDO_C8S0_*vs.* IDO_C5S3_ and IDO_C4S4_. Analysis was also performed for one variant where all cysteines were mutated into serines (IDO_C0S8_) and another variant where all cysteines were mutated into alanines (IDO_C0A8_). Residue distance between r85 and r129 was measured using Mol* Viewer (RCSB PDB).^40^

### Galectin 3 binding affinity

Affinity of IDO_C4S4_-Gal3 for lactose was determined using affinity chromatography in an AKTA Pure chromatography system (GE Life Sciences) equipped with consumer-packable glass column (GE Life Sciences) packed with α-lactose agarose affinity resin (Sigma-Aldrich). Proteins were eluted with a linear gradient of β-lactose (Sigma-Aldrich) in phosphate buffer.

### Secondary structure

Protein secondary structure characteristics were measured using Fourier-transform infrared (FT-IR) spectroscopy (PerkinElmer, USA) and circular dichroism (CD). CD was recorded on a Chirascan™-plus qCD (Applied Phosophysics) with a quartz cuvette of path length 1 mm from wavelengths between 190 and 260 nm with 2 nm intervals. Each sample contained purified protein at ∼0.1 mg mL^−1^ and both sample and baseline contained 10 mM phosphate, pH 7.4, and 140 mM KF. Samples were scanned 5 times with the average spectra reported. For FT-IR, wild-type IDO_C8S0_, IDO_C5S3_, and IDO_C4S4_ in distilled water were lyophilized from frozen. Samples were transferred onto the attenuated total reflectance (ATR) stage and then powder samples were compressed by pressure device to give uniform contact on the ATR crystal. The absorbance spectrum was measured over the range of 900–4000 cm^−1^. The baseline of each spectrum was corrected using the Baseline Correction function within the PerkinElmer Spectrum software. Absorbance at 1650 cm^−1^ represents amide I band, while absorbance at 1550 cm^−1^ corresponds to the amide II band. Secondary structure comparisons were made from the peak shape and intensity of amide I and II bands.

### Imiquimod-induced psoriasis

All studies were conducted with the approval of the University of Florida Internal Animal Care and Use Committee, in compliance with the United States Public Health Service policy on Humane Care and Use of Laboratory Animals. An inflammatory mouse model, imiquimod-induced psoriasis,^7,41^ was used to confirm therapeutic efficacy of IDO-Gal3 mutants. After shaving and removal of remaining hair with depilatory cream, 62.5 μg of 5% imiquimod cream (Patterson Veterinary Supply, cat. num. 07-893-7787), a potent inflammatory agent, was applied to the backs of female C57/BL6J mice (8 weeks old, Jackson labs, PBS: *n* = 8, all others: *n* = 4). Mice were clinically observed and scored daily using a modified psoriasis area and severity index (PASI) score to determine disease severity. Erythema (redness), scaling, and thickening were scored independently and assigned a score between 0 to 4: 0, none, 1: slight, 2: moderate, 3: marked, 4: very marked. The cumulative score was reported as a measure of the severity of inflammation (scale 0–12). On the first day of clinical score emergence (day 3), five 10 μg doses of IDO_C4S4_-Gal3, IDO-Gal3, or an equivalent volume of PBS were injected subcutaneously and evenly distributed across the back. Application of imiquimod cream and clinical scoring was performed every day for a duration of 10 d. Mean clinical scores were compared using a one-way ANOVA and Dunnett's multiple comparisons test. Area under the curve was calculated for each animal, and compared using an unpaired *t*-test. Initial rate of change of scores was measured between the baseline score to plateau for each animal, and compared using an unpaired *t*-test. All statistics were performed using Graphpad Prism.

## Results and discussion

### Cysteine to serine IDO mutants are enzymatically active

**Fig. 1:**
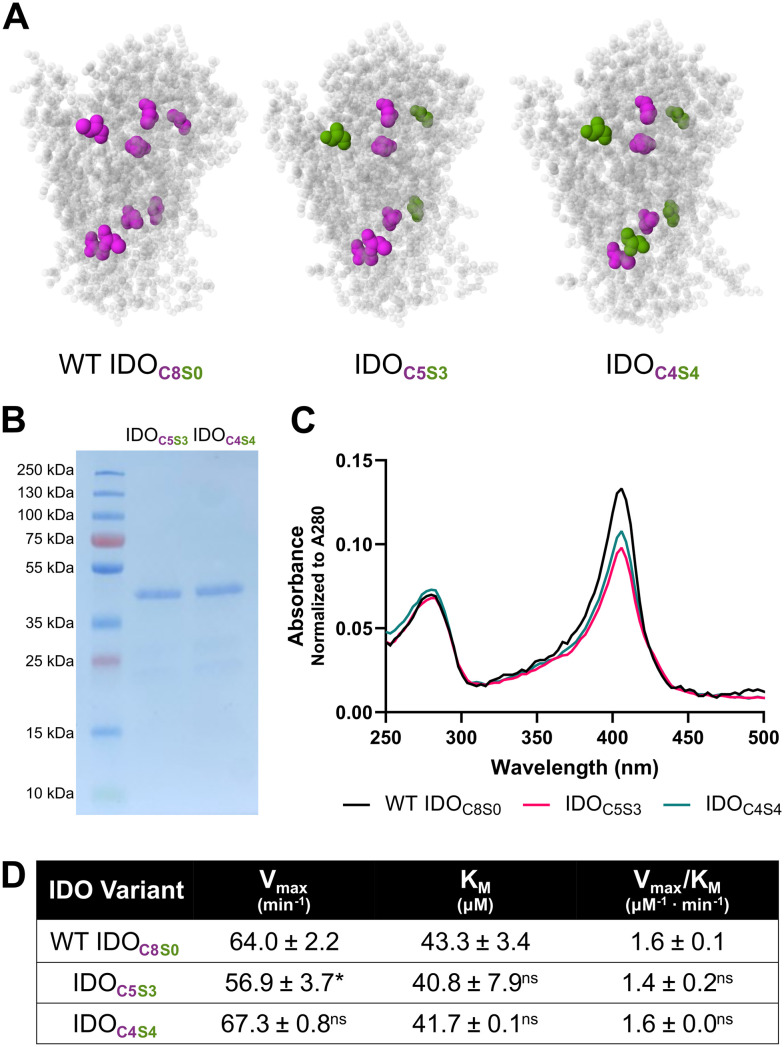
IDO Cys-Ser mutants. (A) Predicted IDO crystal structures highlighting the location of Cys residues in each IDO variant. Cysteines are highlighted in pink while cysteine to serine point mutations are highlighted in green. (B) SDS-PAGE gel of IDO_C5S3_ and IDO_C4S4_ showing a pure product after recombinant expression. (C) Absorbance spectra of WT IDO_C8S0_, IDO_C5S3_, and IDO_C4S4_, with Soret peak around 405 nm. (D) Enzymatic parameters of WT and mutant IDO, compared between mutant and WT. *: *P* < 0.05, ns: no significance.

We designed two different IDO mutants, one where the cysteines located at residues 112, 159, 206, and 308 were substituted for serines, “IDO_C4S4_”, and a second at residues 112, 159, and 308, “IDO_C5S3_” (Fig. 1A). Proteins corresponding to the predicted molecular weights of IDO_C4S4_ and IDO_C5S3_ were recovered in good yield and purity from microbial hosts (Fig. 1B). Soret bands (*λ* = 405–425 nm), which are used to identify the heme unit of IDO,^42^ had similar maxima for WT IDO, IDO_C4S4_ and IDO_C5S3_ (Fig. 1C). Wild-type IDO (“WT IDO_C8S0_”) had a higher Soret band/protein (*λ* = 405/280 nm) peak ratio than that of IDO_C4S4_ and IDO_C5S3_, suggesting that there may be more heme bound to WT IDO. Both IDO mutant forms were enzymatically activity (Fig. 1D). There were no differences in *K*_M_ for either mutant relative to WT IDO; however, IDO_C5S3_ had significantly slower *V*_max_ value than WT IDO_C8S0_ and IDO_C4S4_. This slower *V*_max_ and weaker Soret band of IDO_C5S3_ are correlative, as heme binding to oxygen is required for the dioxygenase reaction of tryptophan.^17,25^

### *In silico* analysis and *in vitro* secondary structure characterization display a high degree of similarity between WT IDO and cysteine-based mutants

**Fig. 2:**
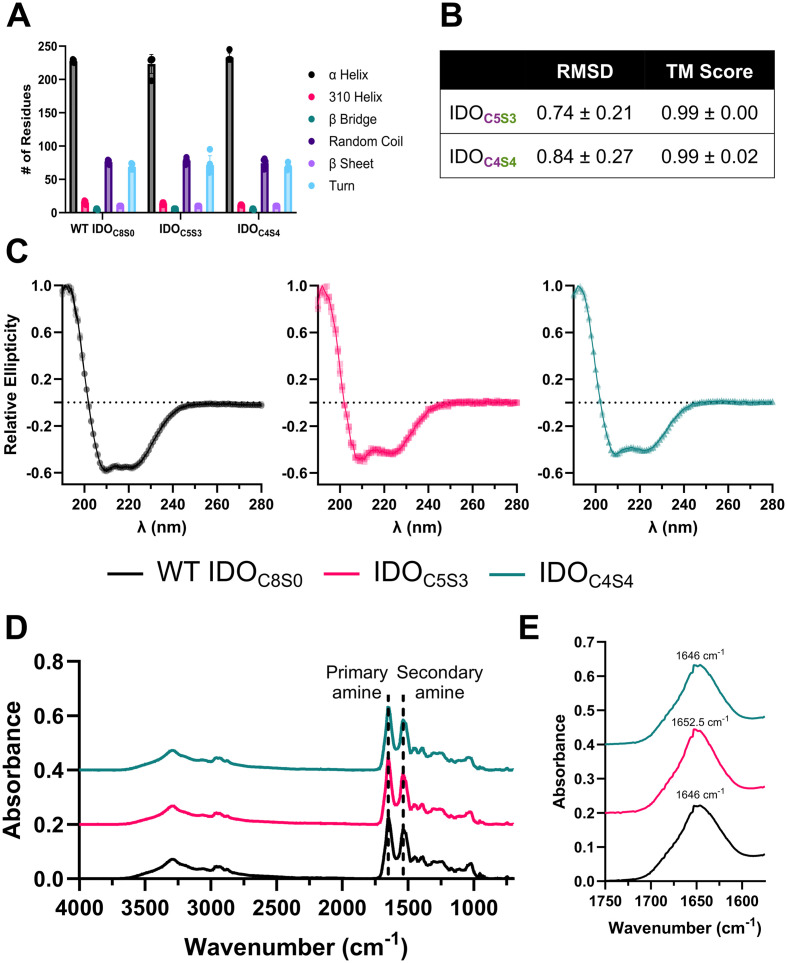
IDO mutants have similar *in silico* predicted and *in vitro* secondary structures. (A) Secondary structure composition comparison and (B) pairwise structural alignment comparison between AlphaFold predictions of WT IDO and IDO Cys-Ser mutants. (C) Circular dichroism spectra of IDO proteins over the range *λ* = 180 to *λ* = 280 nm at 20 °C. (D) Fourier transform infrared spectroscopy (FT-IR) absorbance spectra of IDO and Cys-Ser mutants. Dashed lines at indicate primary and secondary amines. (E) Inset of FT-IR trace, showing primary amine.

Computational models predicted that the Cys-Ser mutations in IDO_C5S3_ and IDO_C4S4_ would not significantly alter secondary structures compared to WT IDO. Composition of predicted secondary structure components showed no significant changes (Fig. 2A), and three-dimensional structural predictions between WT IDO and IDO mutants had a high degree of similarity (Fig. 2B). Likewise, CD and FT-IR measurements showed that the secondary structures of IDO_C5S3_ and IDO_C4S4_ were highly similar to that of WT IDO. The CD spectra of all three proteins were characterized by minima at 208 and 222 nm, along with a maxima at 192 nm (Fig. 2C), consistent with a protein that is predominantly folded into alpha-helices. Additionally, all three proteins demonstrated a prominent peak at 1645–1655 cm^−1^ in FT-IR spectra, indicating a structure predominated by alpha-helical coiled-coils (Fig. 2D), matching characterizations of IDO in the literature.^17,43^ Of note, IDO_C5S3_ displayed a slight blue shift in the maxima of the primary amide band to 1652.5 cm^−1^ (Fig. 2E), which suggests a subtle shift in secondary structure,^44^ and which correlates to the slower *V*_max_ seen in this mutant.

### Cysteine to serine mutants improve IDO thermal stability at physiological and reduced temperatures

**Fig. 3:**
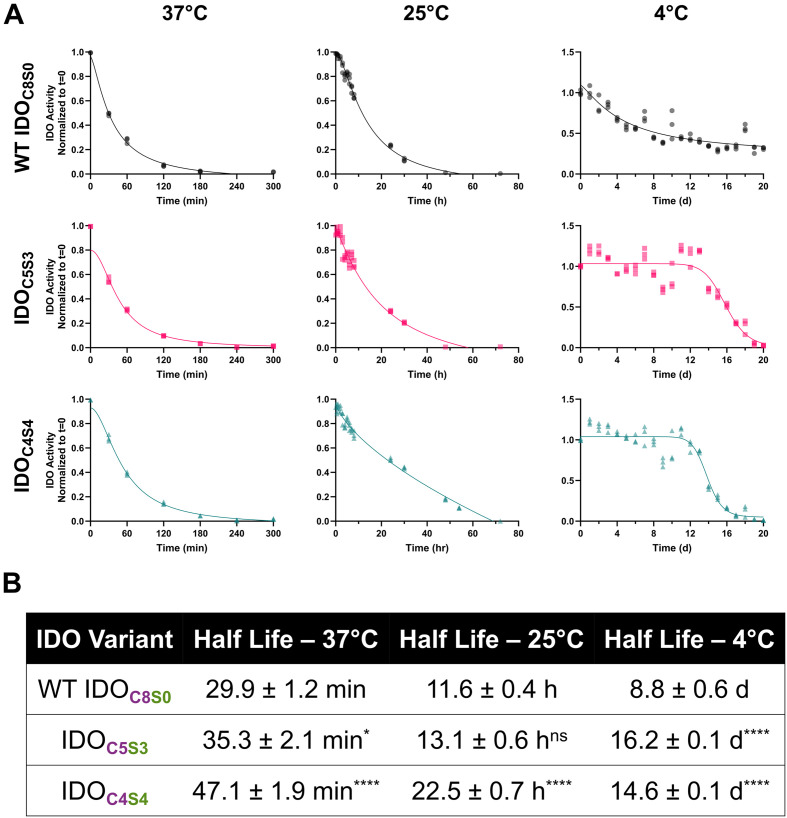
Cys-Ser IDO mutants increase stability of IDO. (A) WT *vs.* IDO_C5S3_*vs.* IDO_C4S4_ normalized enzymatic activity over time after storage at 37 °C, 25 °C, and 4 °C. (B) Mutant IDO activity half-life also increases for all temperatures, as compared to WT IDO_C8S0_. *: *P* < 0.05, ****: *P* < 0.0001.

To assess the effect of the Cys-Ser mutants on thermal stability of IDO, we measured the enzymatic function of WT IDO and the two mutants after warm (37 °C), room temperature (25 °C), and cold (4 °C) storage. Cys-Ser mutants demonstrated higher enzymatic activity over time when compared to WT IDO (Fig. 3A). At physiological temperatures, IDO_C4S4_ extended enzymatic half-life by ∼50% as compared to WT IDO_C8S0_, whereas IDO_C5S3_ only extended stability by ∼20% (Fig. 3B). At room temperature, IDO_C4S4_ increased half-life by ∼200% compared to WT IDO_C8S0_, while IDO_C5S3_ only improved the half-life on the order of hours rather than days (Fig. 3B). Cold storage showed the best improvement in enzymatic stability, as both mutant forms increased the enzymatic half-life by 200% (∼1 week increase) as compared to WT (Fig. 3B). Consistent with this result, cysteine point mutations have been previously reported to improve cold storage and thermostability,^45,46^ suggested to be due to slowed or absent cysteine oxidation.^47^ Such mutations, however, would not be expected to affect the susceptibility of the heme to oxidative degradation, as has been shown for IDO and other proteins.^8,48^

### Cysteine to serine IDO mutations improve therapeutic efficacy in imiquimod-induced psoriasis

**Fig. 4:**
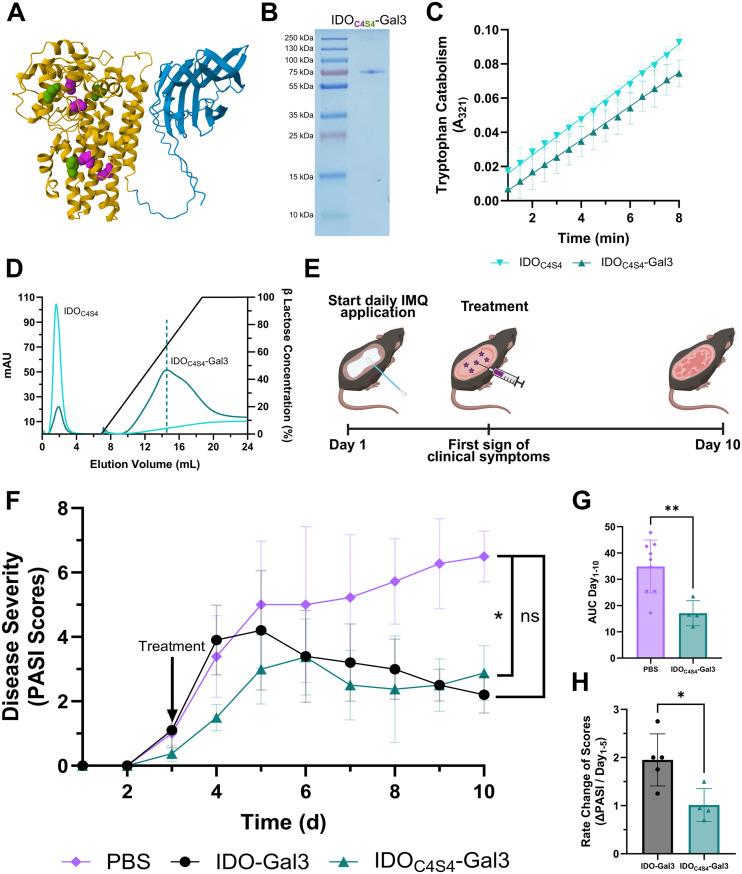
Anchored Cys-Ser IDO mutant improves therapeutic efficacy. (A) Schematic of IDO_C4S4_-Gal3, with the IDO arm shown in yellow and the Gal3 arm shown in blue. Cysteines are highlighted in pink while cysteine to serine point mutations are highlighted in green. (B) SDS-PAGE gel of IDO_C4S4_-Gal3, showing a pure product after recovery. (C) Tryptophan catabolism of IDO_C4S4_ and IDO_C4S4_-Gal3, as shown by absorbance at 321 nm over time. (D) Lactose binding affinity of IDO_C4S4_ and IDO_C4S4_-Gal3, showing preserved galectin 3 functionality in the fusion mutant. (E) Timeline of IMQ-induced psoriasis mouse model. Subcutaneous injection sites are depicted as purple stars. (F) Mutant IDO_C4S4_-Gal3 reduces clinical scores in psoriatic mouse model as compared to PBS treatment, while IDO-Gal3 does not achieve statistical significance of cumulative clinical scores. (G) Area under the curve of clinical scoring is significantly reduced in IDO_C4S4_-Gal3 as compared to PBS. (H) IDO_C4S4_-Gal3 blunts peak of disease and slows rate of inflammation as compared to IDO-Gal3. *: *P* < 0.05, **: *P* < 0.005, ns: no significance.

We created a fusion of IDO_C4S4_ and galectin 3 (Gal3) (Fig. 4A) based on our prior work showing that IDO-Gal3 is effective for treating local inflammation in various diseases.^7,49^ IDO_C5S3_ was excluded from these studies based on its diminished *V*_max_ and weaker thermal stability when compared to IDO_C4S4_. IDO_C4S4_-Gal3 was expressed and recovered in high yield and purity (Fig. 4B). Tryptophan catabolism was comparable between IDO_C4S4_ and IDO_C4S4_-Gal3 (Fig. 4C). The Gal3 domain endowed IDO_C4S4_-Gal3 with binding affinity for immobilized lactose that IDO_C4S4_ lacked, as expected (Fig. 4D).

To test the *in vivo* immunomodulatory capabilities of our IDO Cys-Ser mutant, we used the IMQ-induced psoriasis mouse model, as previously described.^7^ Mice were treated one time with 50 μg subcutaneous injections of IDO-Gal3 or IDO_C4S4_-Gal3, or with an equivalent volume of PBS vehicle control at the first sign of clinical symptoms (erythema, skin thickening, and skin scaling) (Fig. 4E). IDO_C4S4_-Gal3 blunted disease progression of psoriasis (Fig. 4F). Treatment with the mutant led to significantly lower area under the curve for clinical scores relative to vehicle control (Fig. 4G). Treatment with the mutant also delayed peak of disease and slowed rate of inflammation compared to the existing IDO-Gal3 fusion (Fig. 4H). This improved effectiveness of the IDO_C4S4_ mutant corresponded with its increased enzymatic stability and faster *V*_max_.

### Models predict the influence of cysteine mutations to IDO structure

**Fig. 5:**
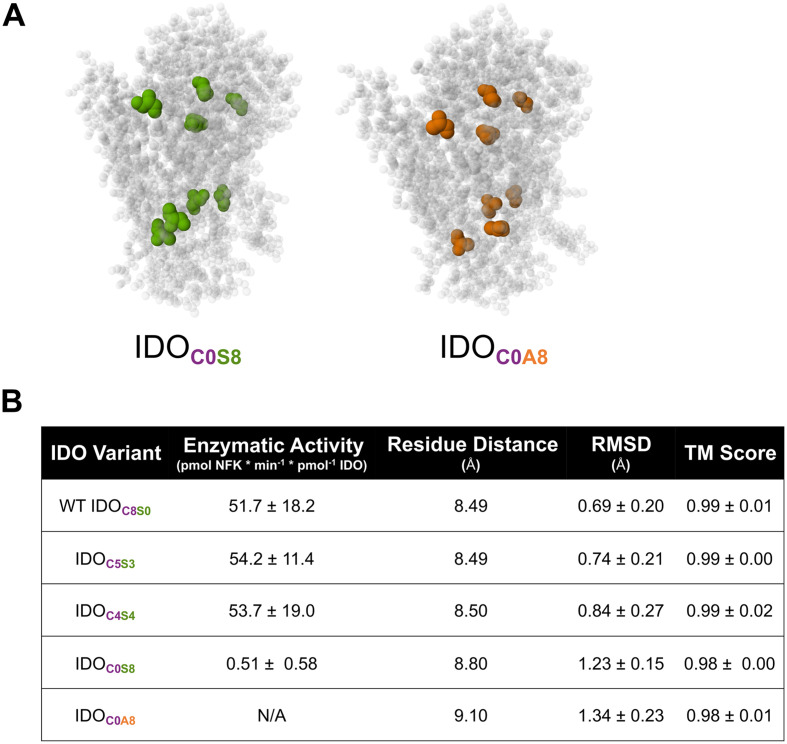
Mutation of cysteines to serines preserves residue distance surrounding enzymatic site. (A) Predicted crystal structures of all Cys-Ser (left) and all Cys-Ala (right) mutations of IDO. Cysteine to serine point mutations are highlighted in green and cysteine to alanine point mutations are highlighted in orange. (B) Activity, predicted distance between site 85 and site 129, and pairwise structural alignment comparison for all mutations.

Modeling of crystal structures predicted that Cys-Ala mutations would change the inter-residue distance between residue 85 and residue 129, which surrounds the enzymatic pocket.^27^ This was suggested to change substrate binding through altered hydrophobic interactions, resulting in a loss of enzymatic activity. Modeling of IDO using AlphaFold v3 predicted that total protein architecture, as measured with RMSD and TM, was not altered in the IDO_C4S4_ and IDO_C5S3_ mutants (Fig. 5B), whereas it was altered in the Cys-Ala mutants reported previously (Fig. 5A). Likewise, AlphaFold v3 models predicted that that inter-residue distances would increase when all Cys were mutated to Ala, IDO_C0A8_, while the selective mutations of IDO_C5S3_ and IDO_C4S4_ were not predicted to increase inter-residue distances (Fig. 5B). Notably, mutating all IDO Cys residues to Ser (IDO_C0S8_) was predicted to significantly increase inter-residue distances, which we expected would lead to reduced activity. Indeed, the IDO_C0S8_ mutant was recovered in poor yield and showed less than 1% activity when compared to IDO_C8S0_, IDO_C4S4_, and IDO_C5S3_ (Fig. 5B). However, even when inter-residue distances were predicted to be similar, as with IDO_C5S3_ and IDO_C4S4_, inserting Ser residues into IDO could have some effect on enzyme activity, as suggested by the slower *V*_max_ for IDO_C5S3_ than for wild-type and IDO_C4S4_, despite IDO_C5S3_ sharing C206 with wild-type, whereas IDO_C4S4_ did not (Fig. 1D). Collectively, these data suggest that while computational models can be used to predict the tolerability of IDO structure to mutations, the impact on enzymatic activity is complex, depending on both the type and placement of the residues, and so must be arrived at empirically.

## Conclusions

The IDO_C4S4_ mutant demonstrated marked improvement in thermal stability and reaction velocity, which provided for a more effective protein therapeutic in the context of psoriasis. Improving reaction velocity and thermal stability of enzymes is often difficult, as protein properties *in vitro versus in vivo* can vary due to many different factors.^50,51^ These improvements were made by mutating surface accessible cysteines to chemically-similar serines, while maintaining cysteines thought to be buried within the fold of IDO. In contrast, prior work using Cys-Ala mutations led to significant decreases in IDO activity.^27^ Collectively, these data reinforce the idea that amino acid choice and site selection are critical for engineering improved mutant enzymes.

## Author contributions

J. A. S., A. W., B. G. K., and G. A. H. conceived of this study. J. A. S., A. W., and A. S. conducted protein production and enzymatic activity assessment. R. A. C. and J. P. conducted secondary structure characterization. J. A. S. performed computational modeling and animal experiments. J. A. S. performed data analysis and wrote the draft of the manuscript. J. A. S., B. G. K., and G. A. H. finalized the manuscript. All authors approved the submitted version of this paper. Artificial intelligence, such as ChatGPT, did not write the manuscript or generate figures outside of AlphaFold v3 predictions, which have been disclosed.

## Conflicts of interest

There are no conflicts to declare.

## Supplementary Material

ME-010-D5ME00106D-s001

ME-010-D5ME00106D-s002

ME-010-D5ME00106D-s003

ME-010-D5ME00106D-s004

## Data Availability

Supplementary information (SI): amino acid sequences. See DOI: https://doi.org/10.1039/d5me00106d. Amino acid sequences can be found in the supplement.
