# Supplementary material for: Engineering an indoleamine 2,3-dioxygenase immunotherapy via selective cysteine-to-serine mutations
Source: Mol Syst Des Eng. 2025 Sep 19;10(12):1090–8. doi: 10.1039/d5me00106d (PMC12498130; doi:10.1039/d5me00106d)
Supplement: ME-010-D5ME00106D-s002 [file ME-010-D5ME00106D-s002.pdf]

Supplementary Information (SI) for Molecular Systems Design & Engineering.
This journal is © The Royal Society of Chemistry 2025

IDO

MGSSHHHHHHSSGLVPRGSHMAHAMENSWTISKEYHIDEEVGFALPNPQENLPDFYNDWMFIAKHLPDLIESGQLR
ERVEKLNMLSIDHLTDHKSQRLARLVLGCITMAYVWGKGHGDVRKVLPRNIAVPYCQLSKKLELPPILVYADCVLANWK
KKDPNKPLTYENMDVLFSFRDGDCSKGFFLVSLLVEIAAASAIKVIPTVFKAMQMQERDTLLKALLEIASCLEKALQVFHQ
IHDHVNPKAFFSVLRIYLSGWKGNPQLSDGLVYEGFWEDPKEFAGGSAGQSSVFQCFDVLLGIQQTAGGGHAAQFLQ
DMRRYMPPAHRNFLCSLESNPSVREFVLSKGDAGLREAYDACVKALVSLRSYHLQIVTKYILIPASQQPKENKTSEDPSKL
EAKGTGGTDLMNFLKTVRSTTEKSLLKEG

IDOC4S4

MGSSHHHHHHSSGLVPRGSHMAHAMENSWTISKEYHIDEEVGFALPNPQENLPDFYNDWMFIAKHLPDLIESGQLR
ERVEKLNMLSIDHLTDHKSQRLARLVLGCITMAYVWGKGHGDVRKVLPRNIAVPYSQLSKKLELPPILVYADCVLANWK
KKDPNKPLTYENMDVLFSFRDGDSSKGFFLVSLLVEIAAASAIKVIPTVFKAMQMQERDTLLKALLEIASSLEKALQVFHQ
IHDHVNPKAFFSVLRIYLSGWKGNPQLSDGLVYEGFWEDPKEFAGGSAGQSSVFQCFDVLLGIQQTAGGGHAAQFLQ
DMRRYMPPAHRNFLSSLESNPSVREFVLSKGDAGLREAYDACVKALVSLRSYHLQIVTKYILIPASQQPKENKTSEDPSKL
EAKGTGGTDLMNFLKTVRSTTEKSLLKEG

IDOC5S3

MGSSHHHHHHSSGLVPRGSHMAHAMENSWTISKEYHIDEEVGFALPNPQENLPDFYNDWMFIAKHLPDLIESGQLR
ERVEKLNMLSIDHLTDHKSQRLARLVLGCITMAYVWGKGHGDVRKVLPRNIAVPYSQLSKKLELPPILVYADCVLANWK
KKDPNKPLTYENMDVLFSFRDGDSSKGFFLVSLLVEIAAASAIKVIPTVFKAMQMQERDTLLKALLEIASCLEKALQVFHQ
IHDHVNPKAFFSVLRIYLSGWKGNPQLSDGLVYEGFWEDPKEFAGGSAGQSSVFQCFDVLLGIQQTAGGGHAAQFLQ
DMRRYMPPAHRNFLSSLESNPSVREFVLSKGDAGLREAYDACVKALVSLRSYHLQIVTKYILIPASQQPKENKTSEDPSKL
EAKGTGGTDLMNFLKTVRSTTEKSLLKEG

IDOC0S8

MGSSHHHHHHSSGLVPRGSHMAHAMENSWTISKEYHIDEEVGFALPNPQENLPDFYNDWMFIAKHLPDLIESGQLR
ERVEKLNMLSIDHLTDHKSQRLARLVLGSITMAYVWGKGHGDVRKVLPRNIAVPYSQLSKKLELPPILVYADSVLANWK
KKDPNKPLTYENMDVLFSFRDGDSSKGFFLVSLLVEIAAASAIKVIPTVFKAMQMQERDTLLKALLEIASSLEKALQVFHQ
IHDHVNPKAFFSVLRIYLSGWKGNPQLSDGLVYEGFWEDPKEFAGGSAGQSSVFQSFDVLLGIQQTAGGGHAAQFLQ
DMRRYMPPAHRNFLSSLESNPSVREFVLSKGDAGLREAYDASVKALVSLRSYHLQIVTKYILIPASQQPKENKTSEDPSKL
EAKGTGGTDLMNFLKTVRSTTEKSLLKEG

IDOC0A8

MGSSHHHHHHSSGLVPRGSHMAHAMENSWTISKEYHIDEEVGFALPNPQENLPDFYNDWMFIAKHLPDLIESGQLR
ERVEKLNMLSIDHLTDHKSQRLARLVLGAITMAYVWGKGHGDVRKVLPRNIAVPYAQLSKKLELPPILVYADAVLANWK
KKDPNKPLTYENMDVLFSFRDGDASKGFFLVSLLVEIAAASAIKVIPTVFKAMQMQERDTLLKALLEIASALEKALQVFH
QIHDHVNPKAFFSVLRIYLSGWKGNPQLSDGLVYEGFWEDPKEFAGGSAGQSSVFQAFDVLLGIQQTAGGGHAAQFL
QDMRRYMPPAHRNFLASLESNPSVREFVLSKGDAGLREAYDAAVKALVSLRSYHLQIVTKYILIPASQQPKENKTSEDPS
KLEAKGTGGTDLMNFLKTVRSTTEKSLLKEG

IDO-Gal3

MAHAMENSWTISKEYHIDEEVGFALPNPQENLPDFYNDWMFIAKHLPDLIESGQLRERVEKLNMLSIDHL
TDHKSQRLARLVLGCITMAYVWGKGHGDVRKVLPRNIAVPYCQLSKKLELPPILVYADCVLANWKKKDPN
KPLTYENMDVLFSFRDGDCSKGFFLVSLLVEIAAASAIKVIPTVFKAMQMQERDTLLKALLEIASCLEKA
LQVFHQIHDHVNPKAFFSVLRIYLSGWKGNPQLSDGLVYEGFWEDPKEFAGGSAGQSSVFQCFDVLLGIQ
QTAGGGHAAQFLQDMRRYMPPAHRNFLCSLESNPSVREFVLSKGDAGLREAYDACVKALVSLRSYHLQIV
TKYILIPASQQPKENKTSEDPSKLEAKGTGGTDLMNFLKTVRSTTEKSLLKEGGS**GGGSGGSGGSGG**EF**A
DNFSLHDALSGSGNPNPQGWPGAWGNQPAGAGGYPGASYPGAYPGQAPPGAYPGQAPPGAYPGAPGAYPG
APAPGVYPGPPSGPGAYPSSGQPSAPGAYPATGP**YGAPAGPLIVPYNLPLPGGVVPRMLITILGTVKPNA
NRIALDFQRGNDVAFHFNPRFNENNRRVIVCNTKLDNNWGREERQSVFPFESGKPFKIQVLVEPDHFKVA
VNDAHLLQYNHRVKKLNEISKLGISGDIDLTSASYNMILEHHHHHH

IDOC4S4-Gal3

MAHAMENSWTISKEYHIDEEVGFALPNPQENLPDFYNDWMFIAKHLPDLIESGQLRERVEKLNMLSIDHLTDHKSQRL
ARLVLGCITMAYVWGKGHGDVRKVLPRNIAVPYSQLSKKLELPPILVYADCVLANWKKKDPNKPLTYENMDVLFSFRD
GDSSKGFFLVSLLVEIAAASAIKVIPTVFKAMQMQERDTLLKALLEIASSLEKALQVFHQIHDHVNPKAFFSVLRIYLSGWK
GNPQLSDGLVYEGFWEDPKEFAGGSAGQSSVFQCFDVLLGIQQTAGGGHAAQFLQDMRRYMPPAHRNFLSSLESNP
SVREFVLSKGDAGLREAYDACVKALVSLRSYHLQIVTKYILIPASQQPKENKTSEDPSKLEAKGTGGTDLMNFLKTVRSTT
EKSLLKEGGS**GGGSGGSGGSGG**EF**ADNFSLHDALSGSGNPNPQGWPGAWGNQPAGAGGYPGASYPGAYPGQA
PPGAYPGQAPPGAYPGAPGAYPGAPAPGVYPGPPSGPGAYPSSGQPSAPGAYPATGP**YGAPAGPLIVPYN
LPLPGGVVPRMLITILGTVKPNANRIALDFQRGNDVAFHFNPRFNENNRRVIVCNTKLDNNWGREERQSV
FPFESGKPFKIQVLVEPDHFKVAVNDAHLLQYNHRVKKLNEISKLGISGDIDLTSASYNMILEHHHHHH
