# Supplementary figures and images for: Engineering an indoleamine 2,3-dioxygenase immunotherapy via selective cysteine-to-serine mutations
Source: Mol Syst Des Eng. 2025 Sep 19;10(12):1090–8. doi: 10.1039/d5me00106d (PMC12498130; doi:10.1039/d5me00106d)

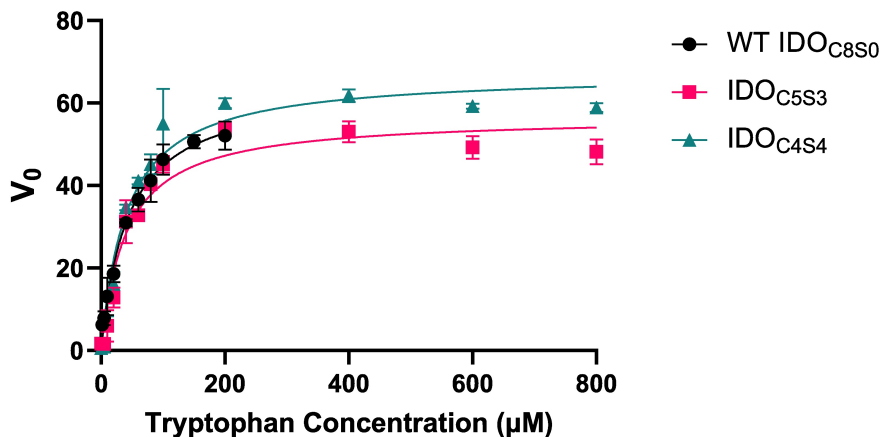

| IDO Variant            | $r^2$ for Michaelis-Menten Curve Fit |       |       |
|------------------------|--------------------------------------|-------|-------|
| WT IDO <sub>C8S0</sub> | 0.959                                | 0.987 | 0.981 |
| IDO <sub>C5S3</sub>    | 0.949                                | 0.963 | 0.928 |
| IDO <sub>C4S4</sub>    | 0.972                                | 0.926 | 0.975 |

Supplement: ME-010-D5ME00106D-s003 [file ME-010-D5ME00106D-s003.pdf]
