# Supplementary material for: Engineering an indoleamine 2,3-dioxygenase immunotherapy via selective cysteine-to-serine mutations
Source: Mol Syst Des Eng. 2025 Sep 19;10(12):1090–8. doi: 10.1039/d5me00106d (PMC12498130; doi:10.1039/d5me00106d)
Supplement: ME-010-D5ME00106D-s004 [file ME-010-D5ME00106D-s004.pdf]

Supplementary Information (SI) for Molecular Systems Design & Engineering.
This journal is © The Royal Society of Chemistry 2025

	Cysteine Residue	V_{max}, Cys-Ala mutant	Oxidative Susceptibility
Solvent Accessible	C112A	59.8 ± 1.5	Yes
	C159A	110.4 ± 2.5	Yes
	C206A	45.5 ± 2.6	Yes
	C308A	94.3 ± 2.4	No
Solvent Inaccessible	C85A	30.6 ± 2.0	Yes
	C129A	101.1 ± 15.0	Yes
	C272A	51.2 ± 0.4	No
	C335A	56.8 ± 0.6	No
